# Prenatal marijuana exposure and visual perception in toddlers: Evidence of a sensory processing deficit

**DOI:** 10.3389/fped.2023.1113047

**Published:** 2023-03-02

**Authors:** Beth A. Bailey, Jahla B. Osborne

**Affiliations:** ^1^ College of Medicine, Central Michigan University, Mt Pleasant, MI, United States; ^2^ Department of Psychology, University of Michigan, Ann Arbor, MI, United States

**Keywords:** marijuana, pregnancy substance use, cognitive development, toddler sensory processing, attention

## Abstract

**Background:**

Research has identified a link between prenatal marijuana exposure and multiple outcomes in children, including cognitive development. Several studies have found specific differences in sensory processing and attention, with visual perception especially impacted in school age children. The current study explored whether this effect is evident at an earlier age, and thus our goal was to investigate the relationship between *in-utero* marijuana exposure and sensory processing capabilities in toddlers. We hypothesized that *in-utero* marijuana exposure throughout pregnancy would specifically predict visual sensory hyperactivity in children as young as 15 months of age.

**Methods:**

Participants were 225 15-month-old children whose mothers were recruited during pregnancy. Substance exposure was prospectively collected and biochemically verified, with marijuana coded as no exposure, 1st trimester exposure only, or exposure throughout pregnancy. The Infant Toddler Sensory Profile evaluated 5 domains of sensory processing (visual, auditory, tactile, vestibular, oral).

**Results:**

Prenatal marijuana exposure throughout pregnancy, but not when limited to the first trimester, predicted a two-fold increased likelihood of scoring in a range indicating high levels of seeking out and potentially over-attending to visual stimulation after controlling for potentially confounding factors including other prenatal exposures. Marijuana exposure was not significantly related to other processing domains.

**Conclusion:**

Results indicate that links previously identified between prenatal marijuana exposure and visual function and attention may already be evident at 15 months of age, and also suggest an impact related to continuous/later pregnancy exposure. Our findings, as well as those from previous studies, all suggest visual processing differences for exposed children, differences that may predict emerging issues with visual attention and habituation. As legalization of marijuana continues to increase, further research is clearly needed to examine specific teratologic effects associated with use during pregnancy.

## Introduction

Marijuana is the most commonly used drug in the United States, with 15.9% of the American population (43.5 million people) self-reporting marijuana use in 2018 (1). Reasons reported for using marijuana include enjoyment, stress relief, and pain relief to name a few (2–6). While marijuana has been shown to reduce pain and improve sleep quality (6), the impact of marijuana use during pregnancy remains unclear. The American College of Obstetricians & Gynecologists (ACOG) advises against the use of marijuana during pregnancy (7). However, it is estimated that at least 4.2% of pregnant women still report using marijuana (8). Reports indicate pregnant women may use marijuana for a variety of reasons, such as nausea and/or anxiety management (8). Studies investigating the association between *in-utero* exposure to marijuana and birth outcomes provide mixed results. For example, Crume et al. only reported increased odds of delivering a low birth weight infant (<2,500 g), but not increased odds of neonatal intensive care (NICU) admission or preterm birth (delivery prior to 37 completed weeks gestation) in a sample from Colorado (9). Conversely, Ko et al. found no association between *in-utero* marijuana exposure and low birth weight using PRAMS data (10), while our research team demonstrated increased risk of low birth weight, preterm delivery, and NICU admission following prenatal marijuana exposure in a multi-state sample (11). Most recently, a meta-analysis by Marchand et al. indicated that pregnant women exposed to marijuana are at increased risk of experiencing preterm birth, delivering a low birth weight infant, and having their infant admitted to the NICU (12).

Prenatal marijuana exposure may also impact cognitive development in offspring. Several longitudinal studies have found increased deficits in several cognitive domains for marijuana exposed offspring, such as attention, language comprehension (13), memory (13, 14), visual perception (13), and visual reasoning (15). Chakraborty et al. detailed enhanced performance on a global motion perception task in children at age four and five who experienced *in-utero* marijuana exposure compared to controls, with dose-dependent effects, a finding that suggests a hyperactivity of visual function in the visual cortex of children exposed to *in-utero* marijuana (16). Studies examining individuals with Autism Spectrum Disorder (ASD), a developmental condition characterized by social impairments and repetitive behavior (17), have found links between visual sensory hyperactivity and overactivity in the visual cortex (18, 19). Additionally, irregular visual sensory responsiveness has been shown to be associated with increased symptoms of Attention-Deficit Hyperactivity Disorder (ADHD) (20), a neurodevelopmental disorder defined by inattention and hyperactive/impulsive behavior (21). Both disorders are thought to embody aspects of attention dysregulation, such as failing to habituate task irrelevant stimuli (22, 23). However, the functional implications of visual sensory hyperactivity in offspring from *in-utero* marijuana exposure are less understood. Therefore, the goal of the present study was to investigate the relationship between *in-utero* marijuana exposure and sensory processing capabilities in toddlers using the Infant/Toddler Sensory Profile (ITSP). We hypothesized that *in-utero* marijuana exposure throughout pregnancy would specifically predict visual sensory hyperactivity in children as young as 15 months of age.

## Methods

### Participants

Study participants were a subset of those who participated in a longitudinal study focused on pregnancy health and child outcomes. Characteristics of the parent study have been described elsewhere (24, 25). Briefly, women were recruited at their first prenatal visit from several prenatal practices in Tennessee and Virginia, some for a pregnancy smoking intervention and others as non-smoking controls. The final prenatal sample (over 95% of those approached for study consent) eligible for inclusion in the current study were those who completed at least two pregnancy research interviews (92%) and gave birth to a newborn who survived to delivery hospitalization discharge at one of the study hospitals (95%). This resulted in 1,063 maternal-child dyads. Of these, 250 were selected for follow-up at child age 15 months based on current age of child and representation of different prenatal exposures. Of the 250 selected, 225 (90%) were located, agreed to participate in the developmental follow-up phase of the study, and completed all components of the 15-month assessment session.

### Procedures

Families were contacted by phone, email, and standard mail 4–6 weeks prior to the child reaching 15 months of age and invited to a research session for developmental follow-up. Families chose time of day for the 2-h session to avoid feeding and nap times, and were provided a small monetary incentive for participation. Transportation was arranged and on-site child care was provided for siblings free of charge as needed. During the session, parents completed demographic, substance use, parenting, and family environment surveys, along with standardized parent-assessments of child health and behavior. Developmental assessments were conducted with the child by a single masters’ level trained examiner blinded to prenatal drug exposure status, who also weighed and measured the child. Consistent order of assessment administration across all participants was observed, with breaks for rest permitted as needed. Both the parent study and the developmental follow-up were approved by the IRB at the affiliated university, and new informed consent was obtained for the 15-month assessment.

### Measures

The primary outcome of interest for the current study was performance on the Infant Toddler Sensory Profile (26). This parent report measure evaluates five domains of sensory processing: visual, auditory, tactile, vestibular, oral. To complete the ITSP, the parent indicates the frequency of the child’s responses (Almost Always, Frequently, Occasionally, Seldom, or Almost Never) to various sensory experiences. Scores can be grouped based on established thresholds. For this study, we grouped total responses on each domain based on these established cut-points to compare those “More than Others” and “Much More than Others” (i.e., greater than one standard deviation above the mean for the norming sample) with all those “Just Like the Majority of Others,” “Less than Others,” and “Much Less than Others” (i.e., equal to or less than 1 standard deviation above the mean). Higher scores indicate seeking out or preferring a high level of stimulation in that domain, and may indicate failure to habituate and over-attention leading to failure to attend to or discriminate other sensory information.

Prenatal substance exposure information was collected prospectively during pregnancy *via* both self-report and biochemical assessment during the initial phase of the study. All participating mothers completed urine drug screens at entry to prenatal care, at least one additional time during pregnancy, and at delivery. Urine drug screens assessed cotinine, marijuana, opioids, benzodiazepines, stimulants, and hallucinogens, and standard laboratory cut-off values were used to indicate positive tests. Additionally, exhaled carbon monoxide levels, a marker of tobacco smoking, were assessed through expired air samples and were considered positive based on established cut-points for pregnant women (27). Women were also asked to self-report any drug use *via* standardized tools including the gold-standard timeline follow-back method (28). Finally, most (93%) newborns had urine drug screens completed on their first urine for all substances listed above, and more than two thirds (71%) had either meconium or cord blood testing for drugs. A woman was considered positive for use of a substance if any of the methods of detection were positive. Additionally, use was classified based on timing during pregnancy when it occurred, with each substance use grouped based on whether a participant used the substance only in the first trimester, or engaged in continued use beyond that. With respect to marijuana, the drug exposure of interest in this study, for those who continued to use past the first trimester, all but one had definitive evidence of still using marijuana at delivery. Thus, use beyond the first trimester was considered to indicate use throughout pregnancy.

Additional data collected included standard demographics and medical history, which were collected *via* maternal self-report throughout the study, and from medical chart review.

### Data analysis

Children who had prenatal exposure to marijuana were compared with those who did not on background factors, including other substance exposure, using *t*-tests and chi-square analysis. Bivariate group differences on the five sensory processing domains were examined using chi square analysis. Analyses controlling for significant background factors and other exposures utilized logistic regression, with odds ratios and 95% confidence intervals reported. All analyses were conducted using IBM SPSS ver 28.

## Results

At the time of the developmental assessment, all children ranged in age from 14 months 2 weeks to 15 months 2 weeks. Of the 225 participants, 65 (29%) had prenatal marijuana exposure, with more than half of these (*n* = 40) exposed throughout gestation.

Comparison of those with and without marijuana exposure on background factors is presented in Table 1. As shown, the two groups differed significantly on several characteristics. Compared to those without exposure, those with *in-utero* marijuana exposure had mothers that were significantly younger with lower levels of education, were less likely to be married and more likely to have a family income below the federal poverty level. In addition, they were significantly more likely to have had prenatal tobacco exposure.

**Table 1:** Participant characteristics by *in utero* marijuana exposure.

	Non-exposed (*n *= 160)	Exposed (*n *= 66)	*p*
Maternal age (years)	26.4 (5.8)	24.4 (5.0)	.011
Maternal education (years)	13.3 (2.5)	12.3 (1.6)	.002
Maternal marital status (% married)	62.9%	33.3%	<.001
Family income (% below federal poverty level)	46.3%	64.2%	<.001
Child age (months)	14.9 (.2)	14.9 (.2)	.993
Child gender (% male)	59.1%	53.1%	.243
Child care (% in any kind of non-parental care)	57.0%	54.5%	.740
Child current second hand smoke exposure (%)	26.3%	28.2%	.626
Prenatal alcohol exposure (%)	7.5%	7.3%	.942
Prenatal tobacco exposure (%)	20.6%	58.5%	<.001

Cell values are mean (standard deviation) or percentage; *p* values are from *t*-test or chi-square analysis.

The relationships between prenatal marijuana exposure and the sensory processing domains are shown in Table 2. *In-utero* marijuana exposure significantly increased the odds of seeking out high levels of visual stimulation, but not when that exposure only occurred during the first trimester. Indeed, exposure to marijuana throughout gestation increased the risk for visual processing differences nearly two-fold after control for potentially confounding factors including gestational exposure to tobacco. Prenatal marijuana exposure did not significantly predict high scores on any of the other sensory domains, although high levels of tactile sensation seeking approached significance in this modestly sized sample.

**Table 2:** Rates and adjusted risk of high level of stimulation seeking by sensory domain following prenatal marijuana exposure.

	No Marijuana exposure	1st Trimester marijuana	Marijuana throughout gestation	Adjusted OR for ANY marijuana exposure^a^	Adjusted OR for marijuana exposure THROUGHOUT gestation^b^
Auditory	28.9%	26.3%	29.8%	.87 (.30–2.58)	1.04 (.51–2.13)
Visual	39.0%	47.4%	55.3%	1.41 (.54–3.66)	1.94 (1.01–3.72)
Tactile	25.2%	27.7%	36.8%	1.14 (.55–2.37)	1.74 (.94–2.71)
Vestibular	37.1%	31.6%	38.3%	.78 (.28–2.17)	1.05 (.54–2.06)
Oral	13.2%	10.5%	17.0%	.77 (.17–3.59)	1.35 (.55–3.78)

Percentages represent rate of those who scored more than 1 standard deviation above the mean, considered to engage in those behaviors “More than Others” or “Much More than Others”. Odds ratios adjusted for maternal age, maternal education, maternal marital status, family income, and prenatal tobacco exposure.

^a^ Reference group is those unexposed to marijuana during gestation.

^b^ Reference group is those unexposed to marijuana or exposed only in the first trimester.

## Discussion

The purpose of our study was to examine the relationship between *in-utero* marijuana exposure and sensory processing capabilities in toddlers using the ITSP. Our prediction, that *in-utero* marijuana exposure (throughout pregnancy) would specifically predict visual sensory hyperactivity in children as young as 15 months of age, was supported. Overall, we had three notable findings. First, we demonstrated (as others have) that pregnant women exposed to marijuana during pregnancy differ in many ways from non-exposed pregnant women. Specifically, pregnant women with marijuana exposure were generally younger in age, less educated, less likely to be married, more impoverished, and more likely to also engage in prenatal tobacco use. This clearly shows the need for extensive control for confounding when examining the impact of gestational exposure to marijuana.

Our second and primary finding was that toddlers who experienced prenatal marijuana exposure were two times more likely to seek out high levels of visual stimulation compared to non-exposed toddlers. We did not find increased odds for any other sensory domain in relation to prenatal marijuana exposure, suggesting specificity to the visual domain. Our third notable finding was that timing of marijuana exposure matters, and that visual processing is primarily impacted when exposure occurs late/throughout gestation. This had implications for intervention, suggesting that significant adverse outcomes related to visual processing may be avoided if pregnant marijuana users cease use by the end of the first trimester.

### Does prenatal marijuana exposure predict a habituation deficit?

Understanding *in-utero* exposure to marijuana in relation to visual sensory processing and visual attention is an important area of study as this relationship might underlie higher level issues in habituation and over-attention to irrelevant information in the external environment. Being able to successfully discriminate between relevant and irrelevant visual stimuli is necessary for daily life functioning in order to focus and avoid external distractions.

Research findings on the relationship between prenatal marijuana exposure and visual sensory processing are mixed. The present study suggests that seeking out high-level visual stimulation begins as early as 15 months of age following marijuana exposure throughout gestation. This finding aligns with previous studies such as that by Chakraborty et al. who uncovered enhanced performance on a global motion perception task for 4–5 year-olds exposed prenatally to marijuana (16), suggesting *in-utero* marijuana exposure is related to overactive visual function. Along similar lines, Leech et al. (29) found evidence for enhanced attention capabilities in 6-year-olds exposed to prenatal marijuana (through the second trimester) based on significantly fewer omission errors on a continuous performance task (CPT)—a common visual attention task (30).

Interestingly, the Ottawa Prospective Prenatal Study (OPPS) reported increased deficits in attention based on significantly more omission errors as measured by a visual attention vigilance task in 5–6 year olds (13). Parental reports from another longitudinal cohort also indicated prenatal marijuana exposure correlates with symptoms of inattention at 10 years old (31), as measured by the Swanson, Noland, and Pelham (SNAP) questionnaire—a common survey used to assess symptoms of inattention and hyperactivity in relation to ADHD (32). Follow-up studies of OPPS participants in adulthood indicate no evidence of executive function deficits, behaviorally, as measured by classic tasks such as the counting Stroop task, which is used to examine susceptibility to distractor interference. However, neurologically, follow-up studies indicated that OPPS participants exposed prenatally to marijuana had significantly more brain activity while completing executive functioning tasks inside a fMRI scanner compared to unexposed controls (33).

Although the research literature appears to be mixed in terms of results, a common theme is that children exposed to marijuana prenatally perform significantly “differently” in some way on tasks of visual perception and attention than those not exposed. In some cases this results in classically “worse” performance, while in others the performance appears to be “better.” This may not be as contradictory as it seems, and likely reflects the specific characteristics of the way in which visual perception is assessed in each study. We propose the showcased “better” visual processing performance, especially in the ranges described in the published studies, may be less of an advantage and more of an indication of a habituation deficit. For example, in the current study we found seeking out high levels of visual stimulation to be related to prenatal marijuana exposure at 15 months of age. This significantly increased desire for visual stimulation may underlie issues of habituating irrelevant stimuli. Thus, children exposed to marijuana *in-utero* may have an affinity toward visual stimulation, which in turn may make it difficult to disengage from irrelevant stimuli, or simply discriminate between relevant and irrelevant visual stimuli. Further study grounded in theories of visual processing development is needed to better understand this issue, and to test this proposed understanding and potential relationship between prenatal marijuana exposure and visual attention and habituation deficits.

The current study has many strengths including prospective and detailed assessment of a wide range of variables including substance exposure. However, several limitations are present. First, sensory processing was assessed here *via* parent report, which can be subject to error related to lack of attention to these issues in their own children and item interpretation differences across parents. Second, while at 225 the sample was reasonably sized for a prospective longitudinal study with comprehensive developmental assessment, investigation of timing of marijuana exposure did reduce the number of participants in each of the marijuana exposure groups to fewer than 50. This resulted in statistical power to find only moderately sized or larger effects. Thus, with a larger sample, it is possible that first trimester marijuana exposure may have significantly predicted visual processing differences, or that gestational marijuana exposure may have significantly predicted processing differences in other domains. A third limitation, related to this issue, is the way in which we examined timing of exposure. Given the patterns of use in this sample, we were only able to examine first trimester use only, or use through to delivery. It is unknown whether use that continues beyond the first trimester but ends at some point prior to delivery predicts processing deficits. It is also unclear whether the patterns of use here are actually proxies for amount of use in that those who quit use by the end of the first trimester may actually be using less marijuana or using less frequently. Thus, use in the first trimester may actually predict sensory processing issues if use is at higher levels than what occurred for our first trimester use only participants. Unfortunately, we did not have reliable data on amount and frequency of use for all of our participants. A fourth limitation of the current study is that while many background differences were controlled for, the number and nature of these differences suggests that there may be other ways, not measured in this study, that women who did and did not use marijuana during pregnancy differed. If these differences, such as continued substance use while parenting, impact sensory processing, it is possible that these factors may partially or even wholly explain the relationships found in this study. Related to this point, due to our sample size, we were also unable to examine the potential impact of prenatal marijuana exposure on sensory processing separately for boys and girls given that gender differences in sensory processing are sometimes evident at this age. While this does not diminish our findings of a global effect regardless of gender, it is a potential avenue of exploration for future studies. A final limitation of this investigation is that the study sample was comprised primarily of disadvantaged rural participants from a region spanning only three states. It is uncertain whether the associations we found generalize beyond this population.

In conclusion, this study provides further evidence of visual processing differences in children prenatally exposed to marijuana, effects not present for other processing domains. This study adds to what is known by demonstrating these effects in children as young as 15 months of age, and also suggests that exposure to marijuana throughout gestation, or at least in the latter stages of gestation, is the primary driver for these effects. Finally, this report suggests that previous seemingly contradictory findings on the association between prenatal marijuana exposure and visual processing may be the result of how this outcome is specifically tested, and that all findings of differences may suggest higher level and later emerging issues with visual attention. Thus, this study adds to the growing body of evidence of the harms of gestational marijuana exposure, and provides further support for clinical recommendations for women to avoid marijuana use during pregnancy.

## Data Availability

The raw data supporting the conclusions of this article will be made available by the authors, without undue reservation.
